# Self-thickening mechanism of an amphiphilic polymer during acid rock reaction and performance testing

**DOI:** 10.3389/fchem.2026.1773935

**Published:** 2026-01-20

**Authors:** Ping Yi, Ruoqin Yan, Yulong Liu, Jinhao Gao, Peng Li, Zhiqiang Dang, Xujie Lin

**Affiliations:** 1 Oil and Gas Technology Research Institute of Changqing Oilfield Company, CNPC, Xi’an, Shaanxi, China; 2 National Engineering Laboratory for Exploration and Development of Low Permeability Oil and Gas Fields, Xi’an, Shaanxi, China; 3 Shaanxi University of Science & Technology, Xi’an, China

**Keywords:** acid thickener, amphoteric polymer, high temperature resistance, self-thickening, zwitterionic

## Abstract

Using acrylamide, methacryloyloxyethyl trimethyl ammonium chloride, and 2-acrylamido-2-methylpropanesulfonic acid as raw materials, an amphoteric ionic self-thickening acid ADAM was prepared. The viscosity changes of ADAM during the acid-rock reaction process were studied, demonstrating that the self-thickening behavior of ADAM depends on the concentration of Ca^2+^ in the solution. Specifically, the ADAM acid solution demonstrated a post-reaction viscosity increase of 110 mPa s, representing a rise of 183%. Temperature and shear resistance of the residual acid solutions were examined using the HAKKE MARS IV. After 1 hour of shear, ADAM retained viscosities of 123, 83, and 28 mPa s at 90, 120, and 150 °C, respectively. These values exceed those of the ADC system by 162%, 295%, and 211% under identical conditions. The ADAM formulation exhibits effective self-thickening capability, and its robust temperature and shear stability support its potential for large-scale application in high-temperature carbonate reservoirs.

## 1 Introduction

Acid fracturing is a key method for stimulating carbonate reservoirs (Jain and Mahto, 2015; Zhao et al., 2015; Zhou et al., 2017). The effectiveness of this process largely depends on the acid penetration radius, which is negatively impacted by acid leak-off—the greater the leak-off, the smaller the effective stimulation radius. Conventional acid systems tend to preferentially flow into high-permeability zones, further increasing their permeability while leaving low-permeability zones under-stimulated (Choi et al., 2007; Fu et al., 2025). Consequently, the acid bypasses low-permeability strata, leading to poor acidification effects in these areas (Fredd and Fogler, 1998). To address this issue, self-diverting acid systems have been developed, which rapidly increase in viscosity after reacting with carbonate rock in high-permeability zones, thereby diverting subsequent acid into lower-permeability regions (Guo et al., 2021).

Currently, several chemical process technologies are adopted to enhance acidification effects in heterogeneous oil and gas reservoirs. These techniques include foam acid diversion, viscoelastic surfactant self-diverting acid technology, and *in-situ* cross-linked acid diversion technology (Cohen et al., 2010), (Kalfayan and Martin, 2009). Among these, viscoelastic surfactant (VES)-based self-diverting acids have attracted attention due to their favorable environmental profile and simple formulation with conventional acids (Abdel-Rahem et al., 2005; Boek et al., 2002; Lynn and Nasr-El-Din, 2001). These systems typically employ cationic or amphoteric surfactants (Schramm, 2000). In cationic systems, the increase in pH and rising concentrations of Ca^2+^ and Mg^2+^ during acid–rock reaction shield electrostatic repulsion between surfactant head groups. This promotes a structural transition of micelles from spherical to rod-like or wormlike structures, leading to a sharp increase in viscosity and effective fluid diversion (Al-Sadat et al., 2014). Amphoteric surfactants behave as cations under strongly acidic conditions. As pH rises during the reaction, ionization of anionic groups increases, resulting in a zwitterionic character. The interaction between surfactant molecules and metal ions (mainly Ca^2+^) induces similar micellar growth and viscosity enhancement, thereby redirecting acid flow toward tighter rock zones. Another approach is *in situ* crosslinked acid technology, typically composed of hydrochloric acid (∼15 wt%), acid-soluble polymers (often containing carboxyl groups), crosslinkers, and gel breakers (Gomaa and Nasr-El-Din, 2015; Lynn and Nasr-El-Din, 2001). The viscosity of such systems is highly pH-dependent. As acid is consumed in high-permeability zones and pH rises above ∼2, crosslinking is triggered, drastically increasing viscosity—often exceeding 1000 mPa s—and temporarily blocking the treated zone to divert acid elsewhere. However, the use of metal-based crosslinkers in such systems can lead to precipitation during gel breaking or in high-temperature/H_2_S environments, posing a risk of formation damage (Nasr-El-Din et al., 2002).

In contrast, self-thickening acid systems employ polymers containing hydrophobic groups that enable viscosity enhancement during acid–rock reaction without requiring an added crosslinker. However, most existing self-thickening acids exhibit limited thermal stability and are typically applicable only up to about 120 °C (Ge et al., 2021; Quan et al., 2022; Tang et al., 2021; Zhao et al., 2018), restricting their use in deeper or hotter reservoirs where temperatures may reach 150 °C or higher.

To overcome this temperature limitation, we developed an amphoteric polymer, designated ADAM, synthesized via aqueous solution polymerization using acrylamide (AM), 2-acrylamido-2-methylpropanesulfonic acid (AMPS), and methacryloyloxyethyl trimethyl ammonium chloride (DMC). For comparison, a copolymer ADC was also prepared from AM and DMC alone. Our study demonstrates that the self-thickening behavior of ADAM during acid–rock reaction is primarily governed by Ca^2+^ concentration. The interplay between anionic and cationic groups in ADAM, modulated by Ca^2+^, leads to viscosity increase while also enhancing the temperature and shear resistance of the spent acid—making it a promising candidate for high-temperature carbonate reservoir stimulation.

## 2 Materials and methods

### 2.1 Materials

Acrylamide (AM, CP), methacryloyloxyethyl trimethyl ammonium chloride (DMC, 75%), 2-acrylamido-2-methylpropanesulfonic acid (AMPS), ammonium persulfate (APS, AR), sodium bisulfite (SBS, AR), hydrochloric acid (HCl, AR), calcium chloride (CaCl2, AR), calcium carbonate (CaCO3, AR), anhydrous ethanol (>99.7%) were purchased from Sinopharm Chemical Reagent Co. 2,2-Azobis (2-methylpropylmimicronitrile) dihydrochloride (V-50, 97%), 2,2′-Azobis (2-methylpropanenitrile) (AIBN, 99%) were purchased from Aladdin’s Reagent Network.

### 2.2 Instrumentation

INVENIO Fourier transform infrared spectroscopy, six-speed rotational viscometer (ZNN-D6B) and HAKKE MARSIV rheometer.

### 2.3 Synthesis of ADC and ADAM

**FIGURE 1:**
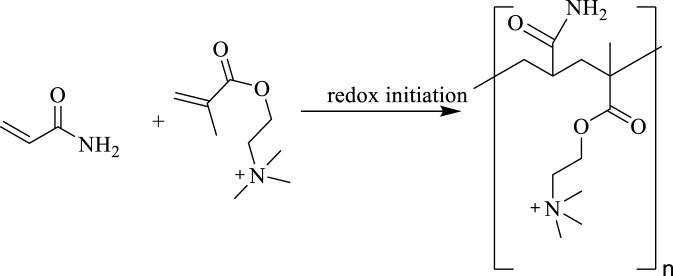
The synthetic route of the ADC molecule.

**FIGURE 2:**
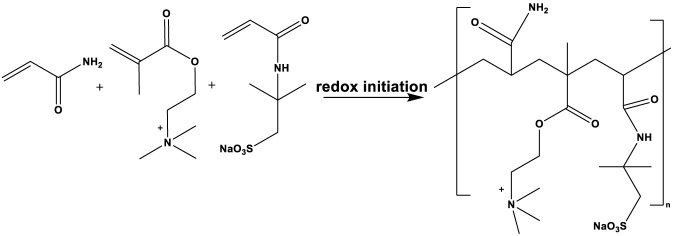
The synthetic route of the ADAM molecule.

The 213 g (3 mol) AM, 104 g (0.5 mol) DMC were dissolved in 683 g of water and fully stirred. The pH of the solution was adjusted to 6 with hydrochloric acid to obtain the ADC reaction solution. The 213 g (3 mol) AM, 104 g (0.5 mol) DMC, and 20.7 g (0.1 mol) 2-acrylamido-2-methylpro-panesulfonic acid (AMPS) were dissolved in 662.3 g of water and fully stirred. The pH of the solution was adjusted to 6 with NaOH to obtain the ADAM reaction solution. The system was cooled to 10 °C using an ice water bath and poured into a thermos flask. After deoxygenation by nitrogen for 30 min, 0.048 g (0.0002 mol) of ammonium persulfate, 0.022 g (0.0002 mol) of sodium bisulfite, 0.5 g of AIBN (0.003 mol), and 0.14 g (0.0005 mol) of V-50 were added, and the reaction was kept at atmospheric pressure for 12 h. After the reaction was completed, the product was granulated by a granulator, dried at 80 °C and ground to obtain the white powder products ADC and ADAM. Purified with ethanol, vacuum drying was performed 24 h. The synthetic routes of the self-thickening agents ADC and ADAM are shown in Figures 1, 2, respectively.

### 2.4 Characterization and performance testing

#### 2.4.1 Characterization

The infrared spectra of ADC and ADAM were measured by the INVENIO Fourier transform infrared spectrometer. The 1H NMR spectra of ADC and ADAM in D2O were characterized by AVANCE NEO 600 MHz.

#### 2.4.2 Rheological properties

The apparent viscosity of 0.7% ADC and ADAM-thickened acids prepared with different concentrations of hydrochloric acid was tested by a six-speed rotary viscometer (ZNN-D6B) at 25 °C and 100 r/min, and the change rule between the apparent viscosity and acid concentration was studied. The temperature resistance, shear resistance, and viscoelasticity of the prepared sample solution were tested by the HAKKE MARSIV rheometer. The conditions for testing thermal and shear resistance are a shear rate of 170s^-1^ and temperatures of 90, 120, and 150 °C, with a heating rate of 0.05 °C/s. After reaching the specified temperature, shear is applied for 1 h to observe the changes in viscosity.

## 3 Results and discussion

### 3.1 Characterization of polymers

#### 3.1.1 FT-IR

**FIGURE 3:**
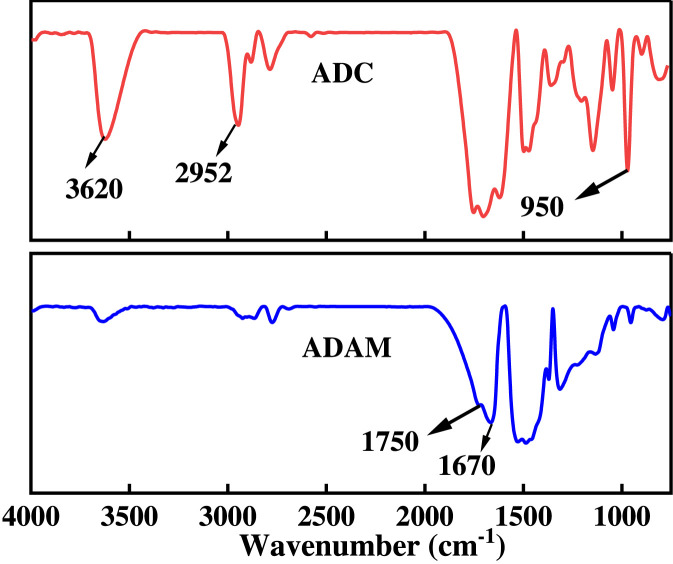
The FT-IR spectra of ADC and ADAM.

Figure 3 shows the FT-IR spectra of ADC and ADAM. Among them, the characteristic absorption peaks at 2,952 cm-1, 1735 cm-1, and 1,130 cm-1 are the stretching vibration peaks of C-H, C=O, and C-O in DMC. The characteristic absorption peaks at 3,620 cm-1 and 1,670 cm-1 are the stretching vibration peaks of N-H and C=O in AM, respectively. 950 cm-1 is the characteristic peak of the bending vibration of-CH2-N(CH3)3 quaternary ammonium group in DMC. Compared with the FT-IR spectrum of ADC, the absorption peak of the FT-IR spectrum of ADAM at 1,670 cm-1 becomes weaker. This is because the electron-withdrawing induction effect of the sulfonic acid group reduces the frequency of the C=O stretching vibration absorption peak. The above proved that ADAM was successfully synthesized.

#### 3.1.2 ^1^H NMR

**FIGURE 4:**
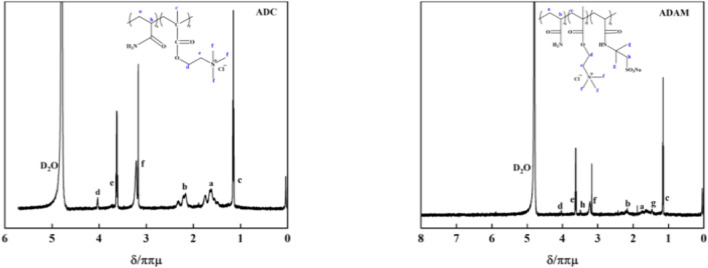
^1^H NMR spectra of ADC and ADAM.

Figure 4 shows the ^1^H NMR spectra of ADC and ADAM. As shown in Figure 4, δ 4.79 is the D_2_O solvent peak; the proton peaks at δ 1.58–1.88 (a) and δ 2.16–2.17 (b) correspond to the hydrogens in the -CH_2_- and -CH- groups on the main chain of the molecule; in the ^1^H NMR spectrum of ADC, the proton peak at δ 1.15 (c) corresponds to the hydrogens in the -CH_3_ group of DMC; the proton peaks at δ 4.03 (d), δ 3.48 (e), and δ 3.17 (f) correspond to the hydrogens in the -O-CH_2_, -CH_2_-N, and -N-(CH_3_)_3_ groups of DMC, respectively; in the ^1^H NMR spectrum of p (ADAM), the proton peak at δ 1.46 (g) corresponds to the hydrogens in the -CH_3_ group of AMPS; the proton peak at δ 3.50 (h) corresponds to the hydrogens in the -CH_2_- group of AMPS. The ^1^H NMR data confirm the successful synthesis of ADC and ADAM.

### 3.2 Rheological properties

#### 3.2.1 Study on thickening mechanism of ADC and ADAM acid solution

ADAM molecular chain contains both C-N(CH_3_)_3_ cationic group on DMC and -SO_3_Na anionic group on AMPS. -SO_3_Na has a good chelating effect on metal cations, which is the main factor affecting the thickening performance of ADAM acid solution. To validate this factor, fresh acid solution, residual acid solution and simulated residual acid solution of ADC and ADAM were configured to test their viscosity.

The 0.7 wt% ADC fresh acid solution was obtained by completely dissolving 1.4 g of ADC in 198.6 g of 20 wt% hydrochloric acid. The residual acid solution with a hydrochloric acid mass fraction of 15%, 10%, 5%, and ∼0% was obtained by completely swelling the 0.7% ADC fresh acid solution and adding 13.7, 27.4, 41.1, and 54.8 g of CaCO_3_ powder according to the reaction relationship between hydrochloric acid and calcium carbonate (molar ratio of 2:1). ADAM was used instead of ADAM to obtain a fresh acid solution of ADAM and a residual acid solution of different acid concentrations. According to the reaction relationship between hydrochloric acid and calcium carbonate (molar ratio of 2:1), a simulated residual acid solution was prepared with CaCl_2_ and different concentrations of hydrochloric acid. For example, during the acid-rock reaction, 15 g (0.411 moL) of HCl was consumed from 100 g of fresh acid (20 wt%) and 22.8 g (0.205 moL) of CaCl_2_ was generated to obtain a 5% residual acid solution. Therefore, a simulated 5% residual acid solution was prepared with 77.2 g of 5% hydrochloric acid and 22.8 g of CaCl_2_. The ratio of hydrochloric acid to CaCl_2_ in the residual acid solution with different acid rock reaction degrees is shown in Table 1.

**TABLE 1:** Configuration of residual acid solution with different acid–rock reaction degrees.

Simulated concentration of hydrochloric acid	20%	15%	10%	5%	∼0%
Addition of hydrochloric acid	100 g of 20% hydrochloric acid	92.47 g of 15% hydrochloric acid	84.94 g of 10% hydrochloric acid	77.2 g of 5% hydrochloric acid	69.88 g of water
Addition of CaCl_2_	0 g	7.53 g	15.06 g	22.8 g	30.12 g

The percentages in the table are mass fractions.

**FIGURE 5:**
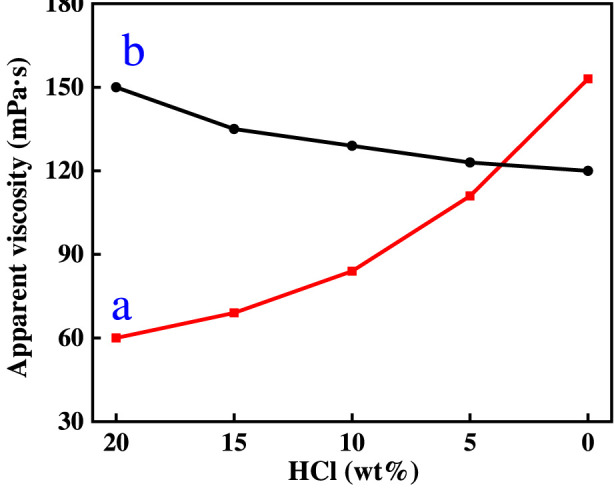
The variation of viscosity of ADC and ADAM acid solution with acid concentration.

Figure 5 shows the change rule of the viscosity of ADC and ADAM two kinds of acid solution, with the acid rock reaction, as can be seen from the figure, the viscosity of ADAM fresh acid solution is 60  mPa s, with the viscosity of ADC fresh acid solution (150  mPa s) is much smaller. This is because in the ADAM molecular chain contains both cationic groups and anionic groups, dissolved in water, the two are attracted to each other, the ADAM molecular chain curls, the viscosity is smaller. However, as the acid rock reaction proceeded, the viscosity of the ADAM acid solution gradually increased (60 mPa s to 153 mPa s), while the viscosity of the ADC acid solution gradually decreased (150–120 mPa s). This is because in the process of acid rock reaction, the concentration of Ca^2+^ in the solution is increasing, for the ADAM molecular chain, Ca^2+^ enters into the solution, because its polarity is stronger than the cationic group in DMC, it is easier to chelate with -SO_3_Na in AMPS, so that the ADAM molecular chain is stretched, and the viscosity of the solution is increased. In contrast, Ca^2+^ enters the ADC acid solution because Ca^2+^ and the cationic groups repel each other, forcing the ADC molecular chains to curl up and the viscosity of the solution to decrease.

**FIGURE 6:**
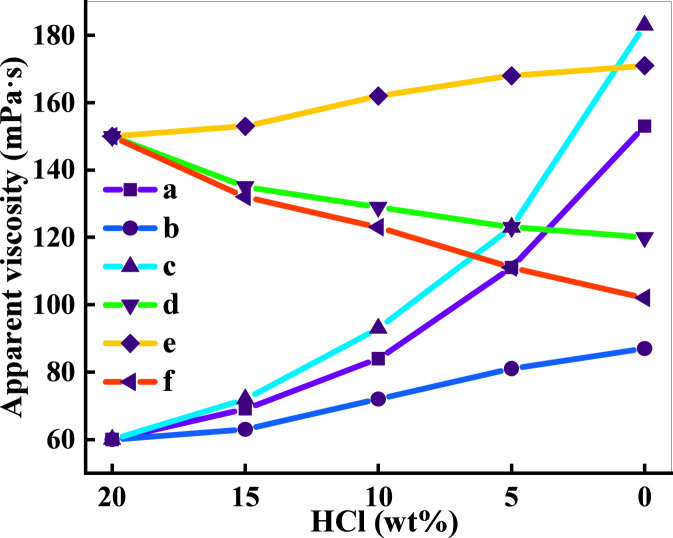
Changing law of viscosity of different acid solutions with acid concentration**(a)** ADAM residual acid solution; **(b)** ADAM prepared solutions with different mass fractions of hydrochloric acid; **(c)** ADAM simulated residual acid solution; **(d)** ADC residual acid solution; **(e)** ADC prepared solutions with different mass fractions of hydrochloric acid; **(f)** ADC simulated residual acid solution.

In order to further verify that the different patterns of change in the viscosity of the two acid solutions, ADAM and ADC, are due to Ca^2+^. ADAM and ADC acid solutions of 0.7% were prepared with different mass fractions of hydrochloric acid, respectively. A 0.7% solution of ADAM and ADC acids was prepared with simulated residual acids prepared with CaCl_2_ and different concentrations of hydrochloric acid. The viscosities of the four solutions were tested. The test results are shown in Figure 6.

**FIGURE 7:**
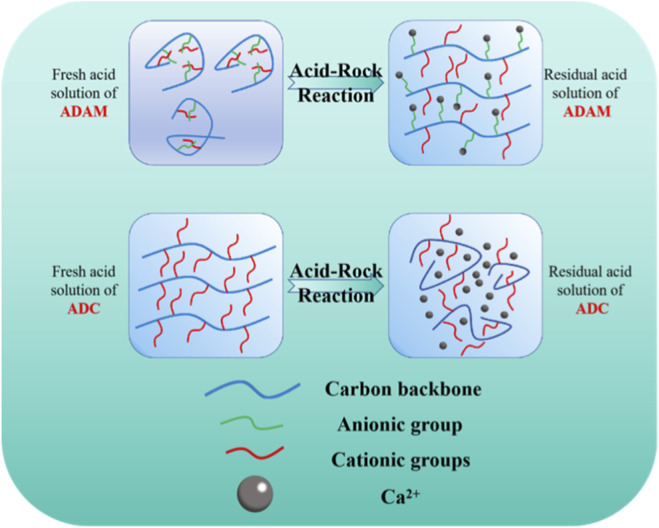
Schematic representation of changes in the state of ADAM and ADC molecular chains as the acid rock reaction progresses.

As can be seen from Figure 6, the viscosity of the three different solutions of ADAM gradually becomes larger with decreasing acid concentration, as shown in the three curves in Figures 6a–c. However, the magnitude of the change in the three curves is different. Comparison of the viscosities of ADAM simulated residual acid solutions and ADAM acid solutions prepared with different mass fractions of hydrochloric acid shows that the simulated residual acid solutions have a higher viscosity than the solutions without the introduction of Ca^2+^ when the acid concentration is reduced to 0% (183 and 87 mPa s, respectively). The viscosity of ADAM acid solutions prepared with different mass fractions of hydrochloric acid increases because the H^+^ in the solution causes the amide group in the ADAM molecular chain to curl up, and the viscosity increasing effect is poor. The acid concentration decreases, the molecular chain stretches and the viscosity increases. Comparison of the final viscosities of the two curves a, c proves that Ca^2+^ is the main reason affecting the viscosity increase of the ADAM acid solution. Comparing the ADAM residual acid solution and the simulated residual acid solution, the viscosity of the simulated residual acid solution is greater than that of the residual acid solution when the acid concentration is reduced to 0%. This is because the residual acid solution is obtained by the reaction between HCl and CaCO_3_, and after the proportional addition of CaCO_3_, the H^+^ in the solution is not fully reacted, and the pH is less than 4, which inhibits the stretching of some of the molecular chains. The pattern of change in viscosity of the three ADC solutions varies with acid concentration. Comparing the three curves, the introduction of Ca^2+^ into the solution makes the viscosity smaller and the absence of Ca^2+^ makes the viscosity larger. This is because Ca^2+^ and the cationic groups in DMC repel each other, causing the ADC molecular chain to curl up and the solution viscosity to decrease. The viscosity increasing principle is shown in Figure 7.

#### 3.2.2 Temperature and shear resistance test

**FIGURE 8:**
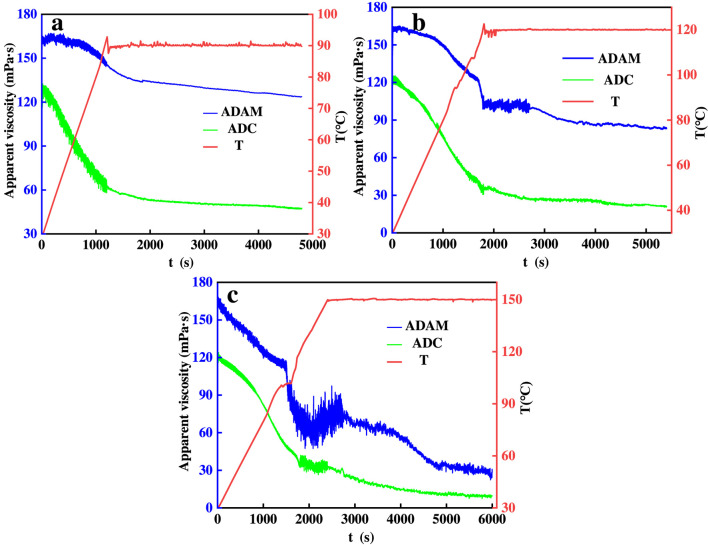
Shear resistance tests of ADAM and ADC residual acid solutions at different temperatures; **(a)** 90 °C; **(b)** 120 °C; **(c)** 150 °C.

As the depth of the reservoir increases, so does the temperature of the reservoir. The more temperature and shear resistant the residual acid solution is, the more it will allow the acid to enter the hypotonic formation and increase the efficiency of acidification. Therefore, the temperature and shear resistance of the residual acid solution is one of the main indicators for evaluating the performance of self-thickening acids. The rheological properties of the residual acid solutions of ADAM and ADC were tested at different temperatures, and the variation rule of viscosity with temperature and time is shown in Figure 8.

As can be seen in Figure 8, the viscosity of the solution continues to decrease with increasing temperature and shear time. Figures 8a shows the shear resistance test of two residual acid solutions at 90 °C. From the figure, it can be seen that the starting viscosity of ADAM is 170 mPa s, and the viscosity is 123 mPa s after shearing at 90 °C for 1 h. In comparison, the residual viscosity of the ADC residual acid solution was 47 mPa s after 1 h of shearing at 90 °C. The viscosity of the ADAM residual acid solution after shearing at 90 °C for 1 h increased by 162% compared to the ADC residual acid solution. In Figures 8b,c, the viscosity of the solution decreases sharply when the temperature is increased to 120 °C, which is caused by the fact that at that temperature the molecular chains start to be destroyed in large quantities (Zhang et al., 2023). The residual viscosities of ADAM residual acid solution after shearing at 120 °C and 150 °C for 1 h were 83 and 28 mPa s, respectively, and the residual viscosities of ADC residual acid solution after shearing at 120 °C and 150 °C for 1 h were 21 and 9 mPa s, respectively, with viscosity increases of 295% and 211%. The temperature and shear resistance of ADAM residual acid solution is stronger than that of ADC residual acid solution, and it is more suitable for acid fracturing modification of high-temperature carbonate reservoirs.

#### 3.2.3 Evaluation of solution viscoelasticity

**FIGURE 9:**
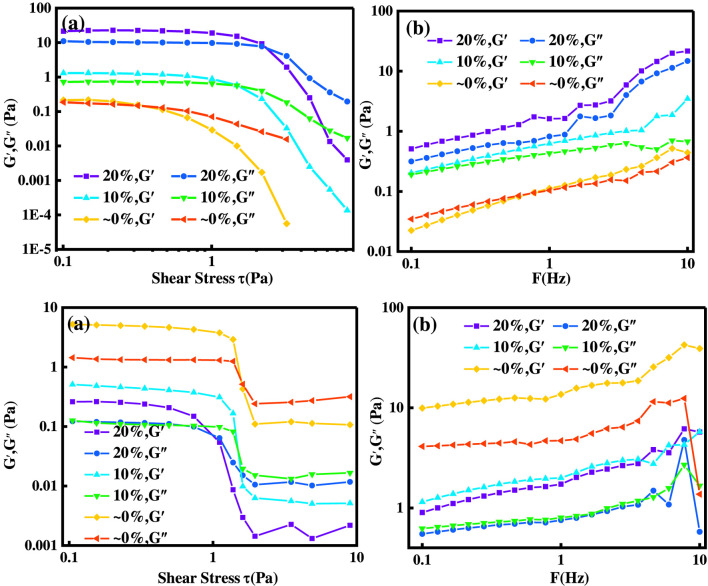
∼10 show the viscoelastic evaluation of ADC and ADAM solutions at different acid concentrations.

As can be seen from the Figure 9, with the progress of the acid-rock reaction, the G′ of the ADC solution is greater than G″, and the solution changes from weakly elastic to viscous. This is because the acid-rock reaction increases the concentration of Ca^2+^, causing the ADC molecular chains to curl. On the other hand, the ADAM solution changes from weakly elastic to elastic, indirectly proving that Ca2+ is the main factor in the self-thickening of ADAM.

The introduction of amphoteric ions enables the self-thickening of ADAM fresh acid solution during acid-rock reactions, because the positive charge carried by Ca^2+^ produced during the acid-rock reaction is stronger than the cationic groups in DMC. Therefore, Ca^2+^ chelates with -SO_3_^-^, releasing the cationic groups in DMC, which increases the viscosity. The starting viscosity of ADAM spent acid solution is higher, and -SO_3_^-^ can provide the polymer with a certain degree of temperature resistance.

## 4 Conclusion

ADC acid liquid thickener was synthesised using AM and DMC as raw materials, on the basis of which ADAM was synthesised by introducing AMPS. ADAM is an amphoteric polymer, and its fresh acid solution can achieve self-thickening phenomenon during the acid rock reaction without adding cross-linking agent. By testing the viscosity of fresh acid solutions, the viscosity of residual acid solutions with different acid concentrations, the viscosity of acid solutions prepared with different mass fractions of hydrochloric acid and the viscosity of simulated residual acid solutions, it was demonstrated that Ca^2+^ is the main factor that enables ADAM amphiphilic polymers to achieve self-thickening during the acid rock reaction. The viscosity of the residual acid solution obtained after the reaction of ADAM fresh acid solution with CaCO_3_ increased by 110 mPa s, or 183%, over the viscosity of the fresh acid solution. Residual acid solutions of ADAM and ADC were tested for temperature and shear resistance using HAKKE MARSIV. At three temperatures, 90, 120 °C and 150 °C, the residual viscosities of ADAM residual acid solution after shearing for 1 h were 123, 83 and 28 mPa s, respectively, which increased by 162%, 295% and 211% compared to the residual viscosities of ADC residual acid solution after shearing. The enhanced temperature and shear resistance of the ADAM residual acid solution and its acid self-thickening ability are expected to be used on a large scale in high-temperature carbonate reservoirs.

## Data Availability

The raw data supporting the conclusions of this article will be made available by the authors, without undue reservation.
